# Mesothelin-targeted immunotoxin RG7787 has synergistic anti-tumor activity when combined with taxanes

**DOI:** 10.18632/oncotarget.13984

**Published:** 2016-12-16

**Authors:** Emily Kolyvas, Michael Rudloff, Marianne Poruchynsky, Rebekah Landsman, Kevin Hollevoet, David Venzon, Christine Alewine

**Affiliations:** ^1^ Laboratory of Molecular Biology, National Cancer Institute, National Institutes of Health, Bethesda, MD, USA; ^2^ Genitourinary Malignancies Branch, Center for Cancer Research, National Cancer Institute, National Institutes of Health, Bethesda, MD, USA; ^3^ Biostatistics and Data Management Section, National Cancer Institute, National Institutes of Health, Bethesda, MD, USA; ^4^ Medical College of Wisconsin, Milwaukee WI, USA; ^5^ Stritch School of Medicine, Maywood IL, USA; ^6^ Department of Pharmaceutical and Pharmacological Sciences, Laboratory for Therapeutic and Diagnostic Antibodies, KU Leuven, Belgium

**Keywords:** mesothelin, immunotoxin, paclitaxel, nab-paclitaxel, pancreatic cancer

## Abstract

Recombinant immunotoxins (RITs) are antibody-based therapeutics that carry a toxin payload. The RG7787 RIT targets the cancer antigen mesothelin to deliver a recombinantly-engineered, reduced immunogenicity variant of *Pseudomonas* exotoxin A (PE) to the cytosol where it inhibits protein synthesis. Here we demonstrate that maximal doses of RG7787 temporarily halt growth of pancreatic cancer tumor xenografts, similar to the approved drugs gemcitabine and nab-paclitaxel, however, combination of the RIT with nab-paclitaxel produces durable complete regressions in most mice. Synergy between taxane and anti-MSLN RITs has been previously demonstrated in mouse models, but direct interaction of the combination in cell culture was not observed. Here, we show that this favorable interaction occurs in cell culture, is dependent on the dose and duration of RG7787 exposure, requires the catalytically active PE, and still occurs with RIT targeting a non-MSLN surface antigen. Unexpectedly, the combination does not increase RG7787-mediated protein synthesis inhibition nor perturb downstream apoptotic markers of RIT-mediated killing, but does augment levels of acetylated tubulin, a marker of taxane activity. Taken together, these data suggest that PE increases cell sensitivity to taxane-mediated killing by increasing taxane-mediated microtubule stability and priming cells for apoptosis by decreasing levels of the pro-survival factor Mcl-1.

## INTRODUCTION

Mesothelin (MSLN) is a cell surface glycoprotein expressed by many solid tumors including malignancies of the lung, pleura, ovary, breast, stomach, bile ducts, uterus and thymus [1]. Expression is particularly robust in pancreatic adenocarcinomas where it can be detected by immunohistochemistry in almost all patient tumor samples [2, 3]. In normal physiology, MSLN is produced only by the mesothelial cells lining the pleura, pericardium and peritoneum and has no expression in the parenchyma of vital organs. The protein is dispensable for normal growth and development and MSLN knockout mice have no phenotype [4]. This strong differential expression pattern makes MSLN an excellent target for antibody-based therapies and several are currently being tested in the clinic [5].

RG7787 is a recombinant immunotoxin (RIT) therapeutic that consists of an anti-MSLN Fab fragment bearing a modified *Pseudomonas* exotoxin A (PE) payload [6]. The anti-MSLN targeting domain binds to the surface of cancer cells, triggering internalization. Inside the cell, PE interrupts the elongation step of translation, halting protein synthesis. This is a unique mechanism of action that is not shared by any currently approved chemotherapies or targeted agents used to treat solid tumors [7]. Pre-clinical studies have demonstrated that RG7787 has picomolar activity against a broad range of tumor cell histologies and can halt or reverse growth of tumors in mice [6, 8, 9]. In addition, RG7787 has substantially decreased toxicity compared to the preceding anti-MSLN RIT, SS1P. The clinical efficacy of previous RITs, like SS1P, has been limited by immunogenicity; most patients develop neutralizing antibodies to the RIT which prevents delivery of effective levels of RIT after initial dosing [10, 11]. RG7787 has been recombinantly engineered to reduce recognition by B and T cells that produce this immune reaction [6, 12]. The success of this technical de-immunization is currently being examined in the clinic (NCT02798536, NCT02810418).

Combining RITs with paclitaxel has been demonstrated to significantly increase anti-tumor efficacy of SS1P and RG7787 in mouse models of multiple tumor types, however, no direct effect of the combination has ever been observed in cells grown *in vitro* [8, 9, 13–15]. In A431 epidermoid cancer cells stably expressing MSLN, initial treatment with paclitaxel increased the amount of SS1P delivered to tumor cells growing in mice [14], but this effect was not observed in a similar model using KLM1 pancreatic cancer cells treated with RG7787 and paclitaxel [9]. The purpose of this study was to examine the interaction of RG7787 with taxanes and determine whether the two drugs directly interact to produces the profound effect of the combination that is observed in mouse models.

## RESULTS

**Figure 1:**
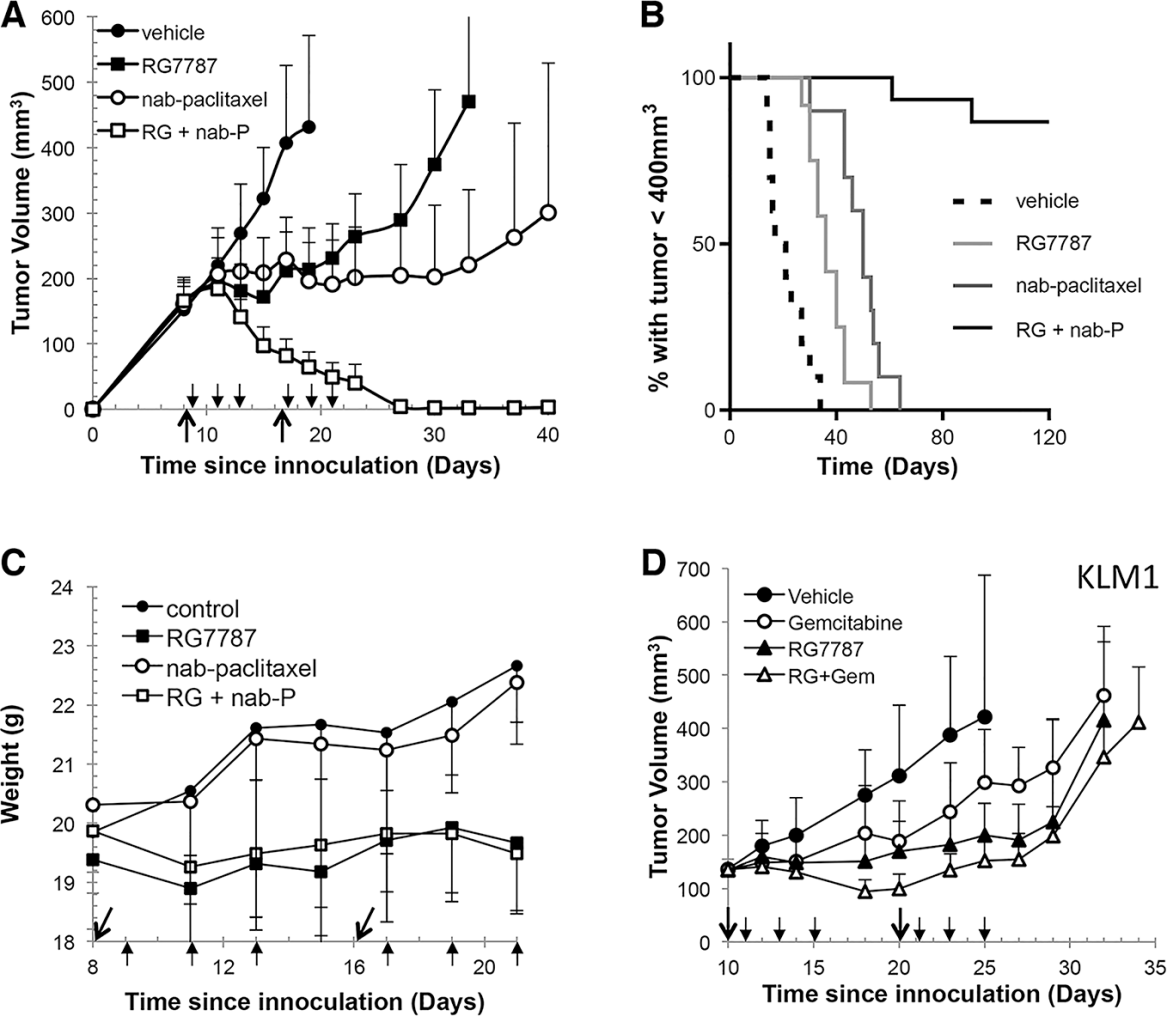
RG7787 produces synergistic anti-tumor responses in combination with nab-paclitaxel (**A**) Athymic nude mice bearing subcutaneous KLM1 tumors were treated with vehicle (*n* = 6), RG7787 (2.5 mg/kg IV qod x3 for 2 cycles, *n* = 8), nab-paclitaxel (100 mg/kg IV x1 for 2 cycles, *n* = 8) or the combination (RG + nab-P, *n* = 8). Results are representative of three experiments. Tumor volume was measured over time. Short arrows designate RG7787 treatment days; long arrows designate nab-paclitaxel treatment days. (**B**) Kaplan-Meier survival curve for mice treated in two different experiments followed for 120 days. Mice were euthanized when tumor volume reached 400 mm^3^. Fourteen of 16 mice treated with the combination did not regrow tumors to this size during the experiment (H.R. for nab-paclitaxel versus combination = 0.057, 95% CI 0.015-0.222). (**C**) Measurement of mouse weight during treatment. (**D**) Mice were treated as above except gemcitabine (80 mg/kg, IP x1 for 2 cycles) was used instead of nab-paclitaxel. Results are representative of two experiments of seven mice per treatment group.

We decided to test the combination of RG7787 with nab-paclitaxel since this taxane is approved for the treatment of pancreatic cancer [16], unlike paclitaxel. First, mice bearing established KLM1 tumors were treated with maximal doses of RG7787, nab-paclitaxel or the combination. Both single agents temporarily halted tumor growth, while treatment with the combination resulted in complete regressions in all of the mice (Figure 1A). These regressions were particularly durable: 88% of mice lacked tumors of even 400 mm^3^ volume at 120 days post-tumor inoculation (Figure 1B). Most mice had no detectable tumor at this time, but three of 13 mice did have reappearance of a mass < 30 mm^3^ between days 60–75 of the experiment. Interestingly, these small masses ultimately regressed or never grew further. We also monitored the weight of mice treated with the combination regimen and observed no increased toxicity compared to single agent treatment with RG7787 (Figure 1C). Nab-paclitaxel was also tested in combination with the LMB-11 immunotoxin, which binds CD-22, a target not expressed by KLM1 cells. Treatment with this combination temporarily halted growth similar to what was seen previously with nab-paclitaxel alone, demonstrating that targeting of the tumor is required for the synergistic effect (Supplementary Figure S1). Next, we tested RG7787 in the same KLM1 model in combination with gemcitabine, another chemotherapy approved for the treatment of pancreatic cancer (Figure 1D). Treatment with the gemcitabine + RG7787 combination slowed tumor growth, increasing time to reach 400 mm^3^ volume from 29.0 days to 34.7 days (*n* = 7, *p <* 0.05). A statistically significant difference in tumor volumes amongst the four groups developed by Day 20 (*p <* 0.001 for one-way ANOVA), with combination treatment decreasing the average tumor volume to 99 mm^3^ compared to 170 mm^3^ for RG7787 alone (post-hoc *t-test p <* 0.05). No complete tumor regressions were observed. These data demonstrate that only selected chemotherapy treatments can induce synergistic efficacy with RG7787 in this model.

**Figure 2:**
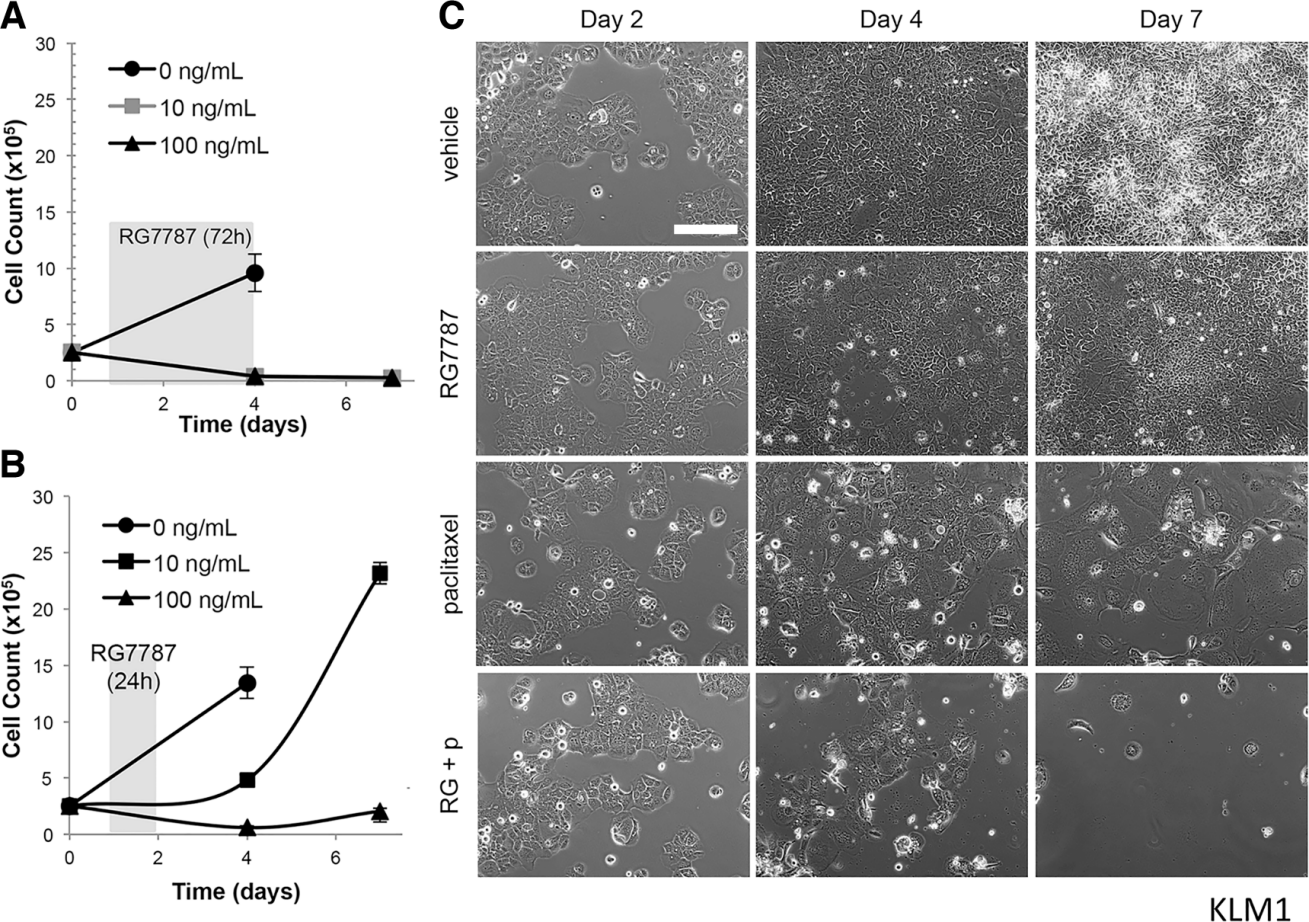
Duration of RG7787 treatment affects anti-tumor efficacy KLM1 cells were treated with the indicated concentrations of RG7787 for 72 (**A**) or 24 hours (**B**) and then triplicate wells were detached and counted at the indicated time points. Shading indicates timing of RG7787 treatment. Data are representative of three or more experiments. (**C**) KLM1 cells were plated on Day 0, treated with vehicle or paclitaxel (6 ng/mL) for 24 hours beginning on Day 1, then treated with vehicle or RG7787(10 ng/mL) for 24 hours beginning on Day 2. Serial photographs of the same field were taken at each of the indicated time points.

Favorable interaction between combinations of mesothelin-targeted immunotoxin and taxanes has also been previously reported for triple negative breast [8], gastric [8], lung [6], mesothelioma (Zhang and Hassan, unpublished observation) and mesothelin-transfected epidermoid cancer cells grown in mouse models as discussed above. Given the profound and consistent effect of this combination we sought to test whether the synergistic anti-tumor activity of the RG7787 and taxane combination could be observed *in vitro*, although previous attempts to do so had been unsuccessful. The IC_50_ for RG7787 in the KLM1 cell line was previously measured to be 4.4 ng/mL (60.9 pM) after a 72 hour exposure [9] and indeed 100% cell kill resulted when KLM1 cells were treated with 10 or 100 ng/mL of RG7787 for this duration (Figure 2A). However, the half-life of RG7787 in mice is approximately 53 minutes (± 5 minutes) for the free drug [6], and mouse tumor uptake experiments have demonstrated that levels of RG7787 begin to fall in KLM1 tumor cells by 12 hours post-treatment [17]. In order to better mimic the conditions prevailing in KLM1 tumors in mice, KLM1 cells in culture were exposed to the same concentrations (0, 10 and 100 ng/mL) of RG7787 for just 24 hours. At 72 hours post-treatment initiation, very few KLM1 cells remained alive at 100 ng/mL of RG7787 treatment. However, KLM1 cell numbers nearly doubled in dishes treated with 10 ng/mL, and by Day 7 the cells had repopulated the dish (Figure 2B). An enhanced effect was observed when cells were treated for 48 hours (Supplementary Figure S2A). This growth metric is similar to our observations of tumor growth *in vivo*. In addition, we have previously demonstrated that dosing cells in culture with 10 ng/mL of RG7787 resulted in similar RG7787 uptake compared to that of the average KLM1 cell in xenografted mouse tumors [17]. For these reasons, the 10 ng/mL dose of RG7787 was selected to use in further experiments.

**Figure 3:**
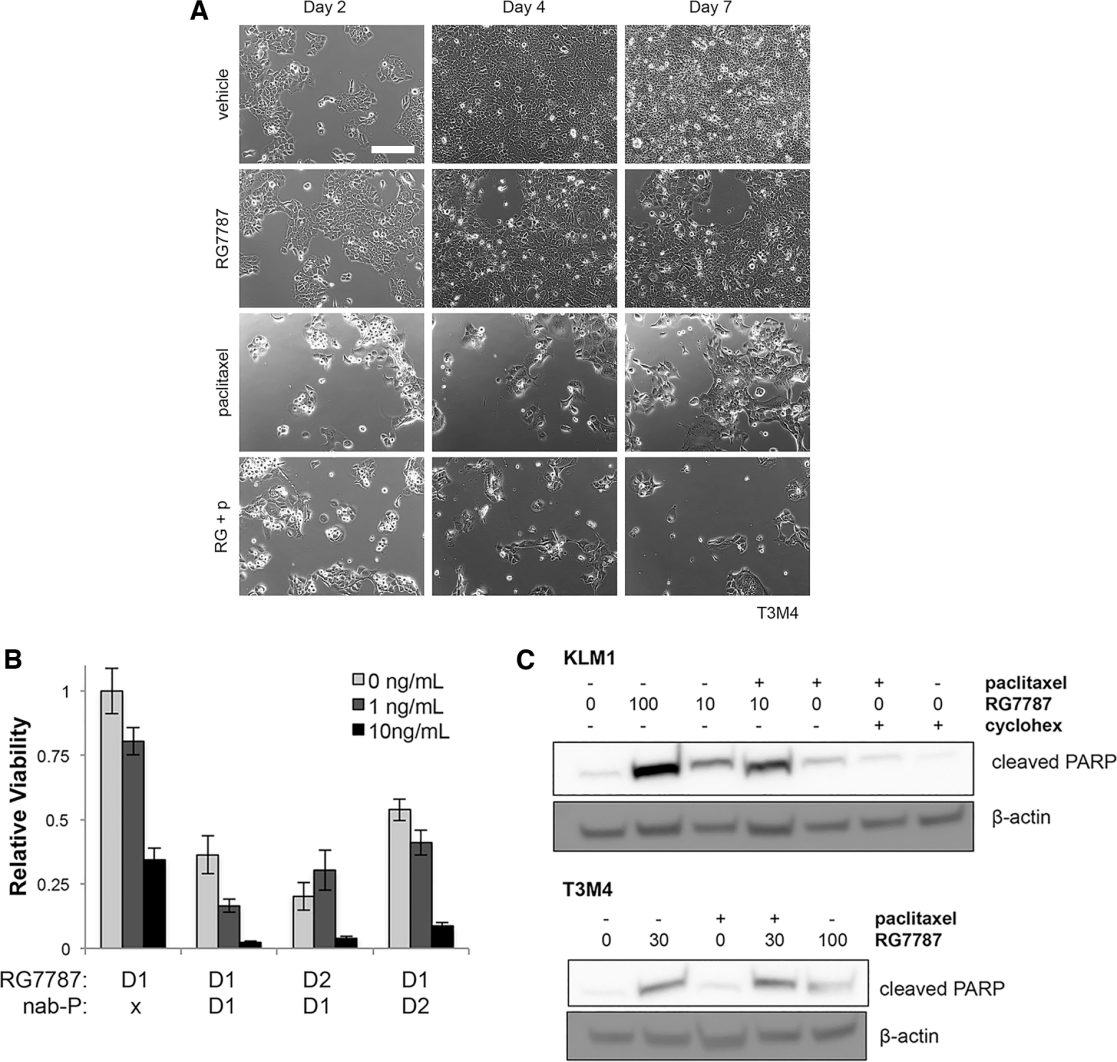
Direct synergism causes increased apoptosis in multiple models (**A**) T3M4 cells were treated and photographed exactly as described in 2C, except that 30 ng/mL RG7787 was used. Images are representative of 3 experiments each performed in duplicate wells. (**B**) KLM1 cells were treated with the indicated concentrations of RG7787 with or without paclitaxel (6 ng/mL) for 24 hours beginning at the time points indicated (D1 = day 1, D2 = day 2). Relative cell viability on Day 7 as assessed by colorimetric assay is shown. Representative of 3 experiments performed in triplicate to sextuplicate. (**C**) KLM1 and T3M4 were co-treated with RG7787 (ng/mL as indicated) and/ or paclitaxel (6 ng/mL) for 24 hours then lysed for protein at 48 hours before immunoblotting. Results confirmed by repeat.

To determine whether synergy could be observed in combination with taxane, KLM1 cells were plated on Day 0 and then treated with vehicle or paclitaxel for 24 hours starting on Day 1 followed by vehicle or RG7787 for an additional 24 hours. Single fields of cells were followed by serial photography. Cells treated with vehicle grew continuously through the course of the experiment, while cells treated with RG7787 or paclitaxel expanded poorly between Days 2 and 4, then rebounded between Days 4 and 7. Almost no combination treated cells remained alive by Day 4 and no regrowth was seen at Day 7 (Figure 2C). In fact, the few remaining cells continued to die over the next 7 days (Supplementary Figure S2B). Similar results were observed when cells were treated with nab-paclitaxel (data not shown), as well as in the T3M4 pancreatic cancer cell model (Figure 3A). No effect was seen when cells were treated with just 1 ng/mL of RG7787 (data not shown). These data demonstrate that the combination of RG7787 and taxanes results in direct enhancement of anti-tumor activity independent of *in vivo* factors such as delivery or metabolism.

To better understand the kinetics of this interaction, the schedule of administration for paclitaxel and RG7787 was altered so that both drugs were given simultaneously, then cell viability was assessed by counting. Co-administration of RG7787 and paclitaxel depleted KLM1 cell numbers and no re-growth was noted by Day 7 (Figure 3B). This schedule resulted in the appearance of increased amounts cleaved PARP by 48 hours post the initiation of treatment with the combination compared to paclitaxel alone in both KLM1 and T3M4 cells, demonstrating that the combination increases apoptotic cell death (Figure 3C). As this simpler schedule appeared to work similarly to giving the two drugs sequentially, subsequent *in vitro* experiments were performed with simultaneous administration of RG7787 and taxane.

**Figure 4:**
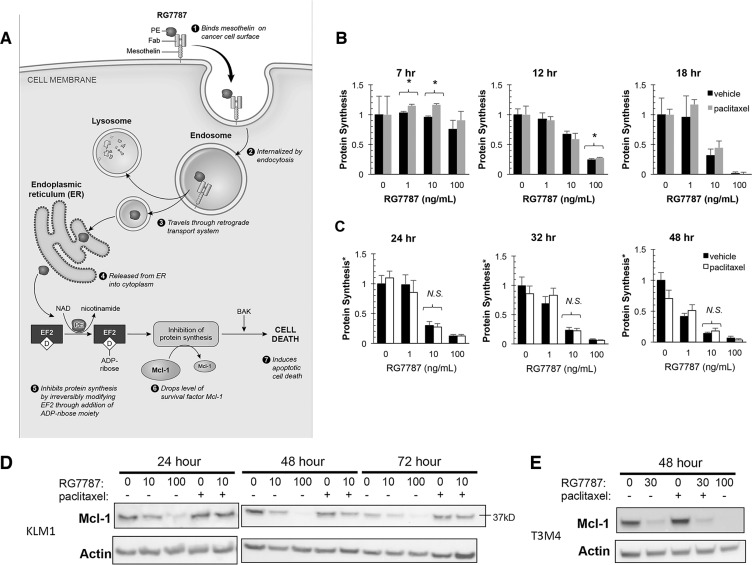
RG7787 enhances cell sensitivity to taxane (**A**) Intoxication model. PE, *Pseudomonas* exotoxin A; NAD, nicotine adenine-dinucleotide; EF-2, Elongation Factor-2; D = dipthamide modification. (**B**–**E**) KLM1 or T3M4 cells were simultaneously treated with the indicated concentrations of RG7787 with or without taxane (paclitaxel or nab-paclitaxel at 6 ng/mL). (B) KLM1 were labeled with radioactive leucine for 2.5 hours starting at the indicated time points and then incorporated radioactivity was measured by scintillation counting. Data presented are averages of 3-5 replicates from a representative experiment. *Indicates *p <* 0.05. (C) Cells were treated with RG7787 ± paclitaxel for 24 hours, then treatment medium was replaced with fresh complete medium for the remaining number of hours indicated before labeling with radioactive leucine. Relative viability of identically treated cells assayed at these time points was concurrently examined using a colorimetric assay. Relative viability measurements were used to correct raw leucine incorporation data to account for differences in cell number caused by differing effects of treatment at these later time points. Data are representative of at least duplicate experiments of 3-6 replicate wells each. (D & E) KLM1 or T3M4 were treated with RG7787 and paclitaxel as described above and then both attached and detached cells were harvested at the indicated time points. Protein lysates were immunoblotted for Mcl-1, a marker of immunotoxin-mediated cell killing. Actin was used as a loading control.

The mechanism of action for RITs has been well described (Figure 4A). RG7787 binds to MSLN on the cancer cell surface, is internalized by endocytosis, undergoes retrograde transport to the endoplasmic reticulum and then is released into the cytosol by an unknown mechanism. In the cytosol, the PE payload catalyzes the irreversible ADP-ribosylation of elongation factor-2 (EF-2). This modification inactivates EF-2 and halts cellular protein synthesis, an insult that leads to apoptosis [7]. Therefore, measurement of *de novo* protein synthesis can be used as a surrogate to assay efficiency of the proximal binding, internalization, processing and trafficking steps in the RG7787 killing pathway. We measured protein synthesis following treatment with RG7787 with or without paclitaxel by quantitating incorporation of ^3^H-leucine at various time points. As expected, a dose-dependent decrease in protein synthesis was seen over time following RG7787 treatment beginning at 12 hours. Maximal protein synthesis inhibition was seen with the 100 ng/mL RG7787 dose by 18 hours post-initiation of treatment (Figure 4B). No recovery of protein synthesis activity was noted even 24 hours beyond the withdrawal of RIT (Figure 4C). Similar results were seen with nab-paclitaxel (data not shown). These data demonstrate that paclitaxel co-treatment must enhance efficacy subsequent to the EF-2 modification step in the intoxication pathway.

Protein synthesis inhibition by RITs preferentially depletes the short-lived Bcl-2 family survival factor Mcl-1 [18]. This alters the balance of pro- and anti-apoptotic factors in the cell, triggering apoptosis. To determine whether taxane treatment can contribute to Mcl-1 depletion, cells were co-treated with paclitaxel and RG7787 for 24 hours then harvested and lysed for protein at the indicated time points. At 24 hours after the start of treatment, 100 ng/mL of RG7787 had already depleted most of the Mcl-1 protein in the cell, the mark of immunotoxin-mediated cell killing (Figure 4D). Paclitaxel alone caused no change in Mcl-1 levels at any time point, nor did its addition to cells treated with 10 ng/mL RG7787 further decrease Mcl-1 levels. Similar results were observed in the T3M4 cell line (Figure 4E). These data suggest that cells treated with the combination die by a different mechanism than those treated with a cytotoxic dose of immunotoxin.

**Figure 5:**
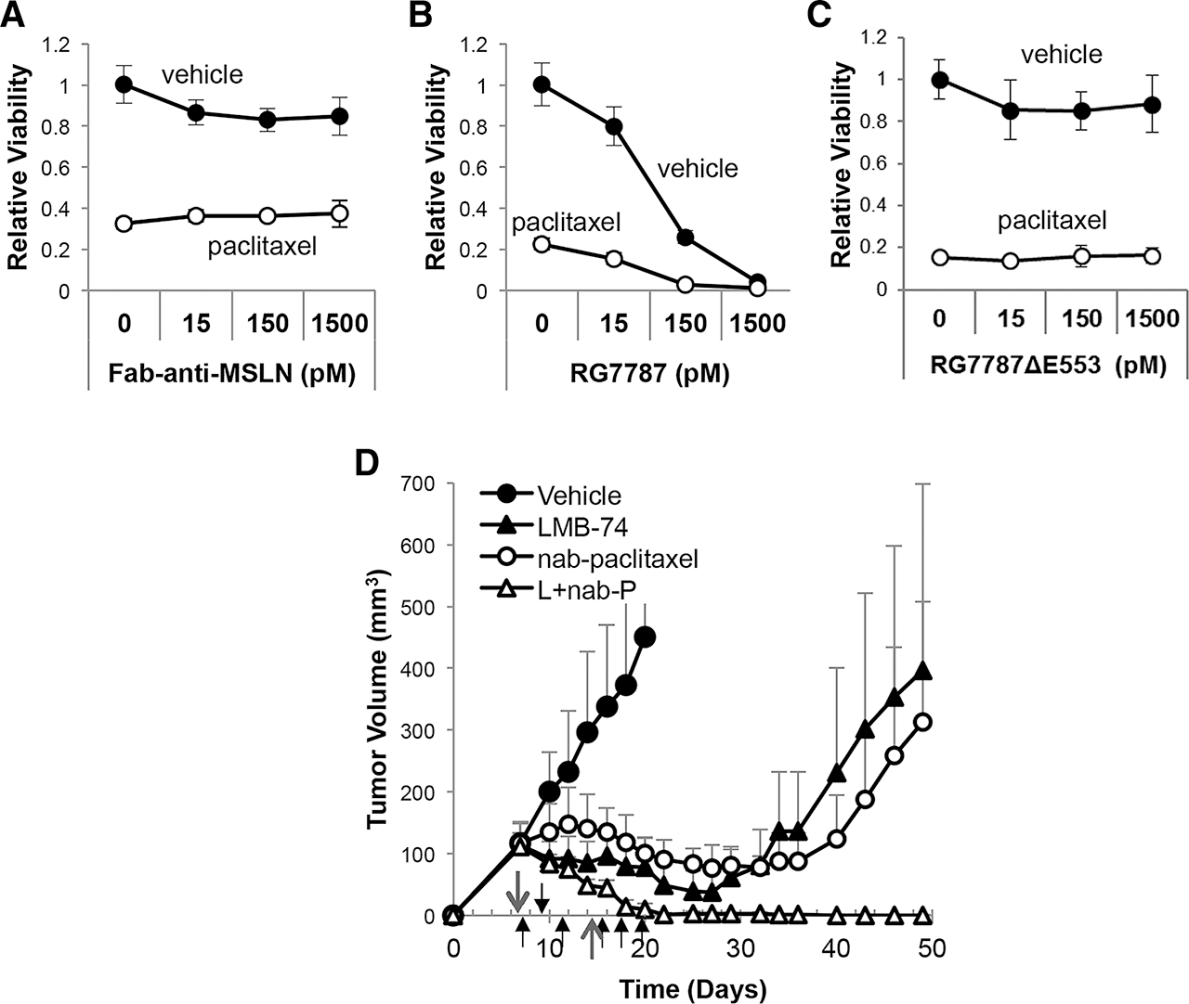
Enhancement of taxane efficacy is not specific to MSLN-targeted toxin (**A**) KLM1 cells were treated with the indicated concentration of Fab-anti-MSLN alone (no conjugated toxin (A)), RG7787 (**B**), or a catalytically dead form of RG7787 which cannot ADP-ribosylate EF-2 (RG7787ΔE553 (**C**)) in combination with (open circles) or without (closed circles) paclitaxel (6 ng/mL) for 24 hours beginning on Day 1. Relative cell viability was assessed by colorimetric assay on Day 7 to quantitate regrowth post-treatment (*n* = 3 wells per group). Results confirmed by repeat. (**D**) Athymic nude mice bearing subcutaneous KLM1 tumor xenografts were treated with vehicle (*n* = 4), the TfR-targeted RIT LMB-74 (L, 0.25 mg/kg IV, qod x3 for 2 cycles, *n* = 8), nab-paclitaxel (nab-P, 100 mg/kg IV, x1 for 2 cycles, n =5) or the combination (L+nab-P, *n* = 8). Tumor volumes were assessed over time. Short arrows designate LMB-74 treatment days; long arrows designate nab-paclitaxel treatment days.

This led us to hypothesize that RIT must enhance the killing efficacy of taxanes. To determine whether the toxin portion of the molecule is required for the increase in paclitaxel efficacy, KLM1 cells were treated with equimolar concentrations of RG7787 or anti-MSLN Fab alone (the binding domain of RG7787) with and without paclitaxel and relative cell viability was assessed 7 days after the initiation of treatment in order to quantitate regrowth. No concentration of anti-MSLN Fab altered cellular regrowth following paclitaxel treatment (Figure 5A), while minimal to no regrowth was seen when 150 or 1500 pM RG7787 was combined with paclitaxel (Figure 5B). This shows that MSLN binding alone is insufficient to induce synergistic killing. Further, treatment with a catalytically dead RG7787 molecule (RG7787ΔE553, Supplementary Figure S3A) resulted in no enhancement of activity in combination with paclitaxel (Figure 5C). This confirms that catalytically active toxin is required for activity.

The RG7787 and SS1P immunotoxins, that have been previously tested in combination with chemotherapy, both target the cell surface cancer antigen MSLN. To determine whether the enhancement of activity seen with RG7787 and taxane combinations is specific to MSLN-targeting, we made a new immunotoxin, LMB-74, with identical structure to RG7787 except that the Fv domains are replaced with sequences derived from the HB21 anti-transferrin receptor (TfR) antibody that confer high affinity binding to the human TfR (Supplementary Figure S3A–S3B). The TfR, like MSLN, is expressed on the surface of KLM1 and other pancreatic cancer cell lines (Supplementary Figure S3C). LMB-74 kills KLM1 and Capan2 pancreatic cancer cells with an IC_50_ in the femtomolar range and produces complete regressions in mice when given at the maximum tolerated dose of RG7787 with no obvious toxicity to the mice (Supplementary Figure S3D–S3E). Once again, co-treatment of RIT in combination with nab-paclitaxel resulted in durable complete regressions using sub-maximal doses of LMB-74 that produce just modest single agent efficacy (Figure 5D). These data show that the synergistic interaction between RIT and taxanes requires the toxin portion of the molecule and still occurs if RIT targets a non-MSLN surface antigen.

**Figure 6:**
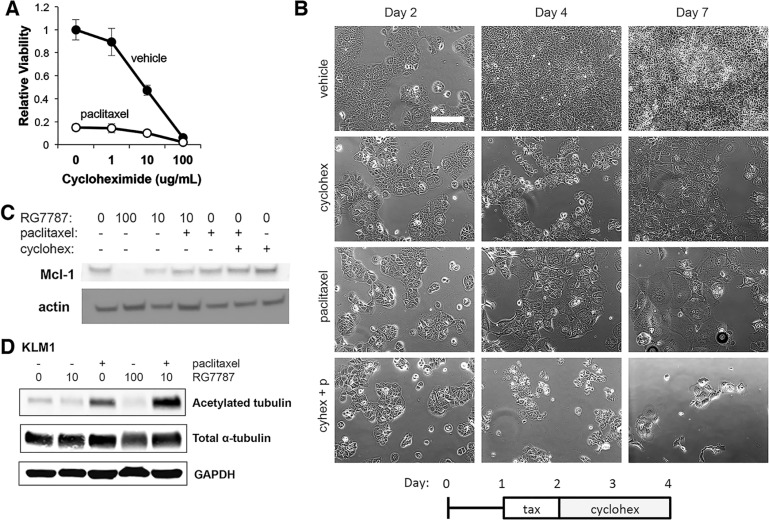
RG7787 primes cells for apoptosis and increases taxane-mediated microtubule stabilization (**A** and **B**) KLM1 cells were treated with paclitaxel or vehicle for 24 hours, then exposed to the indicated concentrations of cycloheximide for 48 hours. (A) Relative cell viability was assessed by colorimetric assay on Day 7 to quantitate regrowth post-treatment (*n* = 5 wells). (B) Cells were serially photographed to visualize viability. Representative of three experiments performed with duplicate or triplicate wells. (**C** and **D**) KLM1 cells were treated with RG7787 (ng/ mL indicated, 24 hours) or cycloheximide (10 mcg/mL, 48 hours) and/or paclitaxel (6 ng/mL, 24 hours) then harvested at 48 hours post treatment initiation. Protein lysates were immunoblotted as indicated. Representative of 3 separate experiments.

PE inhibits protein synthesis even at sub-cytotoxic doses. If enhancement of taxane efficacy depends on the protein synthesis inhibition induced by cytostatic doses of RG7787, then one would expect that an alternative inhibitor of protein synthesis should produce the same synergistic killing effect as RG7787. To test this hypothesis, we treated KLM1 cells with a combination of paclitaxel and the chemical protein synthesis inhibitor cycloheximide. As expected, treatment with cycloheximide for 48 hours produced a dose-dependent decrease in cell populations at Day 7 (Figure 6A–6B). Only the highest concentration of cycloheximide prevented cell regrowth at the Day 7 time point, demonstrating that this concentration was lethal to the cells as a single agent. Addition of paclitaxel to cells treated with vehicle or not fully lethal doses of cycloheximide (0, 1 or 10 mcg/mL), further decreased cell regrowth demonstrating that a cooperative effect of the combination is seen (Figure 5A); however, some cell re-growth did occur, unlike what we observed previously with RG7787 (Figure 6A). Interestingly, treatment with 48 hours of cycloheximide with or without taxane did not reduce Mcl-1 levels nor trigger cleavage of PARP, the latter a marker of apoptosis (Figure 6C and Figure 3C), consistent with this treatment being less efficient at inducing apoptosis.

Apoptotic priming occurs when levels of pro-survival factors are lowered or levels of pro-death factors are increased so that a cell becomes more sensitive to killing by a drug [19]. We have demonstrated here that RIT-mediated protein synthesis inhibition depletes the Mcl-1 survival factor. We subsequently examined how RG7787 and taxane treatment affected levels of additional Bcl-2 family members. No consistent changes in the levels of Bak, Bax or Bcl-xL were observed following 24 hours or 48 hours of treatment (Supplementary Figure S4). However, levels of the pro-survival factor XIAP were affected (Supplementary Figure S4). A small decrease occurred following treatment with taxane alone, but treatment with RG7787 resulted in even lower levels of XIAP, suggesting that RIT treatment may also prime cells for apoptosis by decreasing levels of this survival factor.

Next, we tested whether RG7787 has a direct effect on paclitaxel action. Paclitaxel treatment stabilizes microtubule structures. This decrease in microtubule turnover results in increased acetylation of microtubules. To determine whether co-treatment with RG7787 results in increased taxane-mediated stabilization of microtubules, cells were treated with RG7787 with or without paclitaxel and protein lysates and levels of acetylated-tubulin were determined by immunoblot analysis. A small but reproducible increase in acetylated-tubulin was observed following treatment with the combination compared to paclitaxel alone, while the total level of α-tubulin was not affected by treatment (Figure 6D). This data indicates that immunotoxin treatment increases taxane-mediated stabilization of microtubules.

## DISCUSSION

The taxanes paclitaxel and nab-paclitaxel are microtubule targeted chemotherapy agents that are an important component of standard of care treatment for many solid tumor malignancies including those of the pancreas, lung, breast, ovary, uterus, esophagus, stomach, head and neck [20]. Here, we demonstrate that co-administration of the mesothelin-targeted immunotoxin RG7787 with taxanes can increase the cytotoxicity of incompletely effective doses of taxane and result in durable complete tumor regressions. This effect can be modeled in cell culture demonstrating a direct interaction between the two therapeutic agents. This enhancement is dependent on the administration of a sufficient dose of RG7787 to induce prolonged complete or near complete protein synthesis inhibition, but independent of the surface antigen targeted by the RIT. Previous studies have demonstrated that in some mouse models, pre-treatment with the taxane can boost the amount of RIT delivered to tumor cells. Together these results provide a rationale to test this combination for safety and efficacy in appropriate patient populations.

Our data are consistent with the hypothesis that treatment with RIT increases the sensitivity of tumor cells to taxane-mediated killing. It is difficult to design an experiment to directly prove this hypothesis. Although it is well understood how taxanes interfere with microtubule dynamics, it remains controversial how taxanes elicit cell death. Classically, death is attributed to inability of the cell to exit mitosis, however, previous studies have demonstrated that sensitivity and death of cultured cells in response to taxane treatment do not correlate with duration of mitotic halt [21–23], nor is mitotic halt seen in some susceptible cell types treated with physiologic concentrations of taxane [24]. Lacking a definite readout of taxane killing potency we are left with the preponderance of evidence. First, while lethal doses of RG7787 deplete Mcl-1, treatment with cytostatic levels of RG7787 in combination with taxane do not (just like the single agents), even though all of these cells will go on to die. Secondly, an increase in the amount of acetylated tubulin, a post-translational modification secondary to taxane action, is observed in combination treated cells compared to those treated with taxane alone indicating that taxane treatment produces more stabilization of microtubules under these conditions.

Previous attempts to identify *in vitro* synergy between taxane and RITs have been unsuccessful. The model described here depends upon a critical observation that the duration of exposure to RIT can significantly alter tumor cell response to therapy. Our data indicate that prolonged treatment with low doses of RITs can be as cytotoxic as shorter-term exposure to higher RIT concentrations, meaning that RITs produce both dose- and time-dependent cytotoxicity. For example, a dose of 10 ng/mL can only slow cell growth with 24 hour treatment duration, but is profoundly cytotoxic with a 72 hour exposure. It remains unclear why duration of exposure correlates with response since maximal protein synthesis inhibition is reached within the first 24 hours of therapy and even 24 hours after the halt of therapy no recovery is observed. Nevertheless, this observation suggests that continuous treatment with low doses of RIT may have increased anti-tumor efficacy compared to high dose bolus treatment.

Our previous data in the KLM1 mouse model have demonstrated that bolus dosing of RG7787 results in a peak uptake of RIT by 6 hours, which decreases by 12 hours [17]. Under these conditions, single agent RG7787 halts tumor growth during treatment but does not markedly decrease tumor burden. Nevertheless, RIT treatment does impair the cell's ability to survive taxane treatment. Cytotoxicity from the combination treatment occurs over the course of days, an extraordinarily slow process, that makes classic measures of synergy difficult to apply since cells treated with the single agents reach confluence and cease growing in logarithmic fashion before all combination treated cells finish dying. This prolonged course of cell death suggests that the RIT treatment inhibits the cell's ability to recover from taxane. In summary, our data demonstrate direct synergistic anti-tumor activity of the RIT and taxane combination. RIT directly increases the ability of taxane to stabilize microtubules and also primes cells for apoptosis by decreasing Mcl-1 and XIAP levels. A clinical trial of RG7787 (now renamed LMB-100) in combination with nab-paclitaxel has been initiated (NCT02810418) and uses a treatment schedule based upon the data presented here.

## MATERIALS AND METHODS

### Cell culture and reagents

KLM1 and T3M4 cells were the gift of Udo Rudloff and Mitchell Ho (both of NCI, Bethesda, MD), respectively. Identity was confirmed by STR analysis. Capan2 cells were purchased from ATCC. All cell culture reagents were purchased from Invitrogen except where otherwise noted. All cells were grown at 37°C with 5% CO2 in RPMI 1640 medium supplemented with 2 mmol/L L-glutamine, 100 U penicillin, 100 μg streptomycin and 10% FBS (HyClone, Thermo Scientific). LMB-11 was synthesized as previously described [25]. TP-38 was provided by Darell Bigner (Duke University, Durham, NC) [26]. RG7787 was manufactured by Roche and provided for these studies through a Collaborative Research and Development Agreement. LMB-74 was created by grafting the light and heavy chain variable domain sequence of the HB21 anti-human transferrin receptor antibody into template plasmids that contained the remaining Fab light chain or heavy chain/ toxin fragment (see Supplementary Figure S3A). The immunotoxin was synthesized as previously described [25]. Anti-mesothelin Fab was provided by Di Xia (NCI, Bethesda, MD) [27, 28]. RG7787ΔE553 was provided by Roche. Paclitaxel (Hospira, Inc) was used at a dose of 6 ng/mL freshly diluted in culture media for all experiments.

### *In vitro* photo screen

Cells were plated at a density of 50,000 cells per well in a plate marked with registration grids. Cells were incubated for 24 hours to allow attachment, and then treated with vehicle or paclitaxel (6 ng/mL) for 24 hrs. Medium was gently removed and replaced with fresh medium containing vehicle, RG7787 or cycloheximide. Treatment medium was removed and replaced with fresh medium at the indicated time. Cells from identical fields were photographed at multiple time points using a Zeiss phase contrast microscope using the Axio-Cam MRc camera and the AxioVision 4.7.2 acquisition software.

### Cell counting and *in vitro* quantitative cell viability assay

For counting, triplicate wells of cells were detached with trypsin at the indicated time points and counted with a Cellometer Vision machine (Nexcelom). Relative viability of treated cells was measured using the Cell Counting Kit-8 WST-8 assay (Dojindo Molecular Technologies, Inc.). The reagent was added as per manufacturer's instructions, cells were incubated at 37°C, and absorbance at 450 nm was measured. Values were normalized between 0% viability for treatment with staurosporin or hygromycin positive controls, which produced complete cell killing, and 100% for addition of complete medium alone.

### Measurement of protein synthesis

Medium was removed from treated cells at the indicated time points and replaced with leucine-free RPMI (Sigma) containing 2 uCi/mL ^3^H-Leucine (Perkin Elmer). Cells were incubated at 37°C for 2.5 hours, lysed by freezing on dry ice, thawed, collected on filter mats and then samples were counted using a Wallac Beta plate reader (Boston, MA). Protein synthesis was normalized to that of vehicle treated cells at each time point. All measurements were made in at least triplicate for each experiment.

### Antibodies and immunoblotting

Adherent and detached cells were harvested by scraping, washed in PBS and lysed in RIPA buffer containing protease inhibitors (Thermo Scientific). For studies of tubulin, cells were harvested in lysis buffer (20 mM Tris, pH 7.5, 150 mM NaC1, 1mM EDTA, 1% Triton X-100 with protease and phosphatase inhibitors), frozen and sonicated to clarify. Protein concentrations were determined by Bradford assay using Coomassie Protein Assay Reagent (Thermo Scientific). Samples were separated by SDS-PAGE and transferred to nitrocellulose or PVDF. The following antibodies were used: mouse anti-Mcl-1, rabbit anti-Bak, and rabbit anti-Bax (Abcam); mouse anti-β-actin (Thermo Scientific, BA3R); rabbit anti-cleaved PARP Asp214, rabbit anti-XIAP and rabbit anti-Bcl-XL (Cell Signaling Technologies), mouse anti-human CD71 (transferrin receptor) PE conjugate (Biolegend), mouse anti-acetylated tubulin (Sigma, T7451), mouse anti-a-tubulin (Sigma, T9026), and GAPDH (Millipore, 6C5). HRP-conjugated mouse and rabbit secondary antibodies (Santa Cruz Biotechnologies or Cell Signaling Technologies) were visualized using SuperSignal West Pico Chemiluminescent Substrate (Pierce). Alternatively, IRDye infrared secondary antibodies (LiCor) were utilized and blots scanned on the Odyssey (LiCor).

### Mouse tumor experiments

All animal experiments were performed in accordance with NIH guidelines and approved by the NCI Animal Care and Use Committee. Female 6–8 week old athymic nude mice (Charles River, Frederick, MD) were inoculated subcutaneously with 3–4 × 10^6^ KLM1 cells in 4.0 mg/mL matrigel (Corning). Tumors were allowed to grow to ∼150 mm^3^ then mice were randomized to treatment groups. RG7787, LMB-74 and gemcitabine were diluted in 0.2% human serum albumin. Nab-paclitaxel was diluted in normal saline. Immunotoxins and nab-paclitaxel were delivered intravenously by tail vein injection while gemcitabine was given intraperitoneally. Tumor size was measured in two dimensions by digital calipers and tumor volume was calculated using the formula: *0.4 x width^2^ x length*.

### Statistics

GraphPad Prism and Microsoft Excel were used for all graphing, statistical calculations and curve fitting. Data are presented as averages with error bars marking standard deviation except where otherwise indicated. Two-tailed Student's *t-test* was used for two group comparisons. Comparison of more than two groups was assessed by analysis of variance (ANOVA) with post-hoc analysis by Tukey's adjustment. Tumor regression times were analyzed by the Kaplan-Meier method, and the hazard ratio (HR) was estimated using the log-rank method.

## SUPPLEMENTARY MATERIALS FIGURES


