# Supplementary material for: Mesothelin-targeted immunotoxin RG7787 has synergistic anti-tumor activity when combined with taxanes
Source: Oncotarget. 2016 Dec 16;8(6):9189–99. doi: 10.18632/oncotarget.13984 (PMC5354724; doi:10.18632/oncotarget.13984)
Supplement: Supplementary file 1 [file oncotarget-08-9189-s001.pdf]

Mesothelin-targeted immunotoxin RG7787 has synergistic anti-tumor activity when combined with taxanes

Supplementary Materials

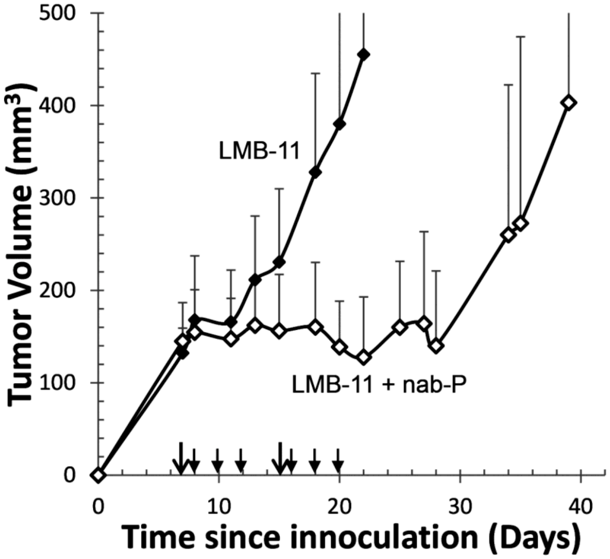

Supplementary Figure S1: Athymic nude mice bearing subcutaneous KLM1 tumors were treated with LMB-11 (2.5 mg/kg IV qod x3 for 2 cycles) with or without nab-paclitaxel (100 mg/kg IV x1 for 2 cycles). Tumors were measured over time. Short arrows designate LMB-11 treatment days; long arrows designate nab-paclitaxel treatment days.

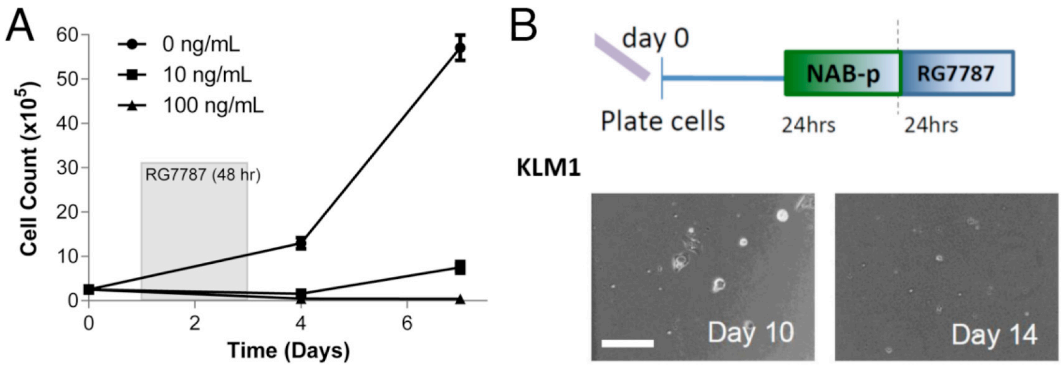

Supplementary Figure S2: (**A**) KLM1 cells were treated with the indicated concentrations of RG7787 for 48 hours then cells from triplicate wells were counted. Results were confirmed by repeat. (**B**) KLM1 cells were treated with nab-paclitaxel (6 ng/mL) for 24 hours on Day 1 followed by RG7787 (10 ng/mL) for 24 hours on Day 2. Cells were followed by serial photography out to Day 14 as indicated. Initial time points were presented in Figure 2C. Cells that remained alive until Day 7 continued to die even at the later time points shown here.

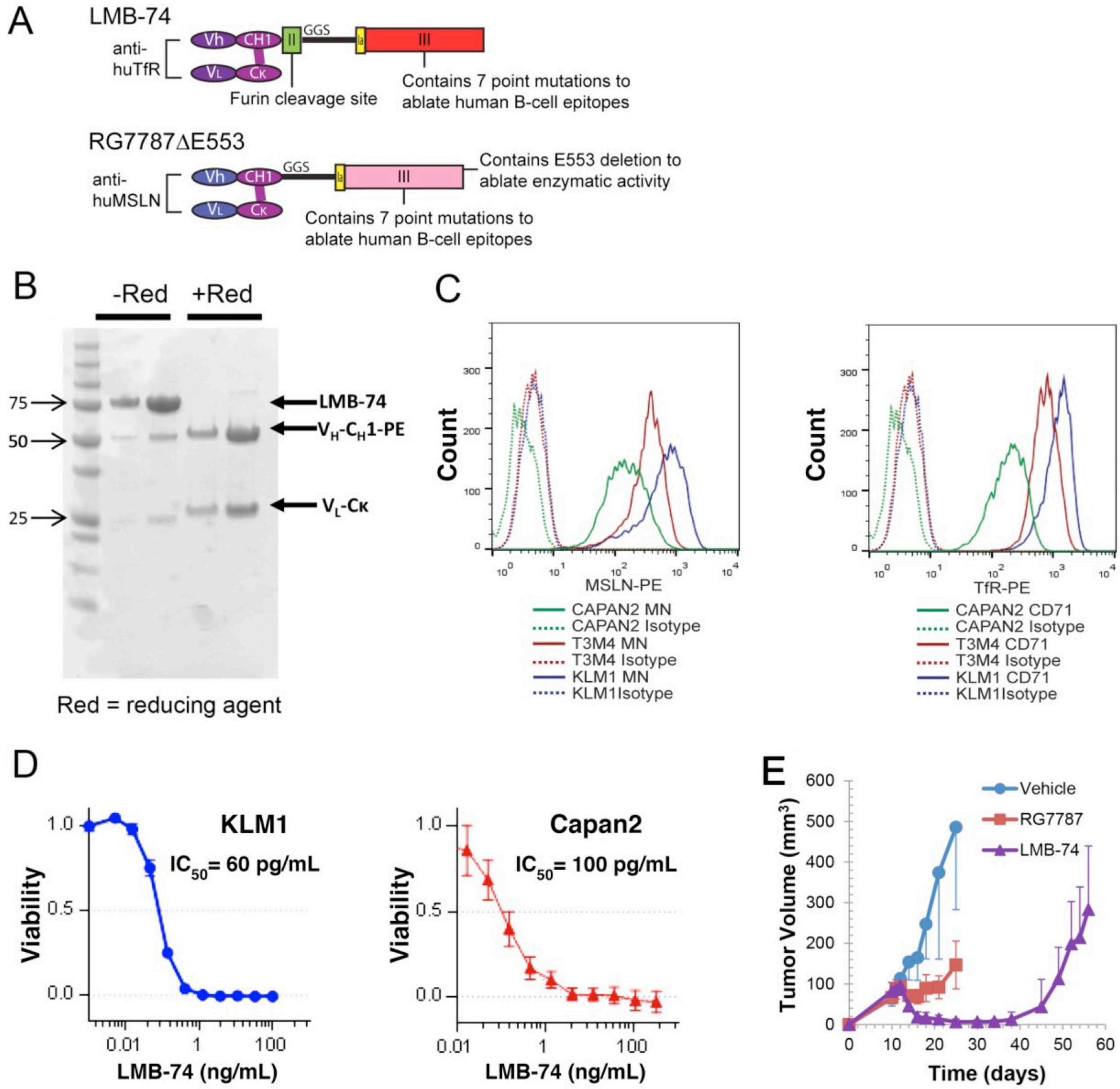

Supplementary Figure S3: (**A**) Structure of TfR RIT LMB-74 and catalytically dead RG7787ΔE553. LMB-74 consists of Fab region which binds TfR. Light chain is bound to heavy-chain-PE chain by disulfide bridge between CH and Vκ. A glycine-glycine-serine (GGS) linker connects the remaining regions of PE domain 2 (II) to the domain 3 catalytic region (III and Ia) of PE. RG7787ΔE553 lacks the furin cleavage site which is critical to RIT processing as well as a the E553 residue which is critical for ADP-ribosylation activity. (**B**) Gel showing the synthesized LMB-74 immunotoxin. (**C**) Flow cytometry examining surface expression of MSLN and TfR in multiple pancreatic cancer cells lines. (**D**) KLM1 or Capan2 cells were treated with various concentrations of LMB-74 for 72 hours and then cell viability was assessed by colorimetric assay. Data is representative of at least three individual experiments. (**E**) Athymic nude mice were inoculated with KLM1 tumor cells on Day 0 and then treated with vehicle (0.2% human serum albumin), RG7787 (2.5 mg/kg) or LMB-74 (2.5 mg/kg) on Days 12, 14, and 16. Tumor volume as measured by digital calipers is shown. No toxicity was observed.

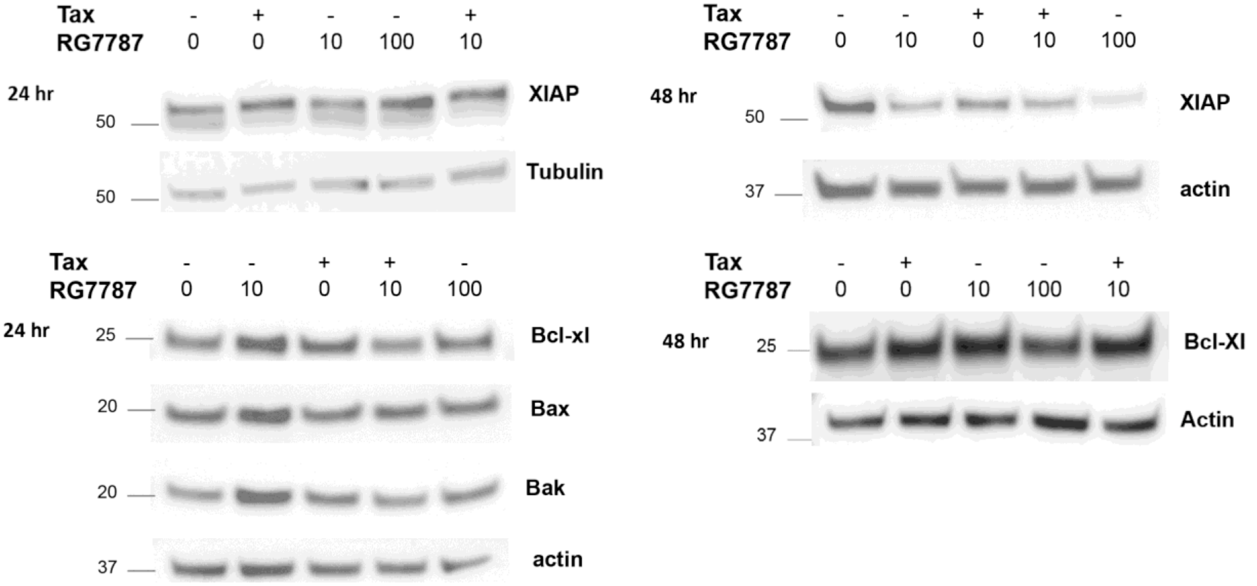

Supplementary Figure S4: Lysates from KLM1 cells treated for 24 hours were immunoblotted for the indicated proteins important in cell survival and apoptosis. Consistent levels of Bak and Bax expression at 48 hours post-treatment were not observed over more than five experiments and were deemed inevaluable at this time point.
